# Hospital variation in the treatment of cT1a renal cancer

**DOI:** 10.1002/bco2.70130

**Published:** 2026-04-09

**Authors:** Cato Caroline Bresser, Paul Bastiaan van der Nat, Hilin Yildirim, Bart Jean Pieter Kersten, Hein Johannes Josephus Leenarts, Katja Karen Helmi Aben, Patricia Jeannelle Zondervan, Lea Magdalena Dijksman, Pepijn Dione Polm, Mirjam Marjolein Garvelink, Harm Hubertus Edmundus van Melick, for the Santeon RCC Working Group, Wouterus Antonius Scheepens, Sybren Dijkstra, Marit Josephine Yska, Daphne Luijendijk, Marino Asselman, Annebeth Evelien Cathelijn Ruiter

**Affiliations:** ^1^ Department of Urology St. Antonius Hospital Nieuwegein/Utrecht the Netherlands; ^2^ Department of Value Improvement St. Antonius Hospital Nieuwegein/Utrecht the Netherlands; ^3^ IQ Health Science Department Radboudumc Nijmegen the Netherlands; ^4^ Department of Research and Development Netherlands Comprehensive Cancer Organisation Utrecht the Netherlands; ^5^ Department of Urology Amsterdam UMC Amsterdam the Netherlands; ^6^ Catharina Hospital Eindhoven the Netherlands; ^7^ Canisius Wilhelmina Hospital Nijmegen the Netherlands; ^8^ Maasstad Hospital Rotterdam the Netherlands; ^9^ Martini Hospital Groningen the Netherlands; ^10^ Medisch Spectrum Twente Enschede the Netherlands; ^11^ OLVG Amsterdam the Netherlands

**Keywords:** cT1a renal cancer, hospital variation, Netherlands Cancer Registry, practice variation, renal cell carcinoma

## Abstract

**Objectives:**

To evaluate treatment patterns and inter‐hospital variation of cT1a renal cell carcinoma (RCC) in seven Dutch teaching hospitals.

**Patients and methods:**

In this historical multicenter cohort study, adults diagnosed with cT1a renal cancer (2019–2022) were identified through the Netherlands Cancer Registry. Clinical data were extracted from electronic records. Primary outcome was initial treatment. Descriptive statistics and subgroup analyses assessed variation. Logistic regression analyses identified factors associated with active treatment.

**Results:**

We included 501 patients with 544 cT1a renal cancer tumours. Mean age was 66 years, 40% were overweight (BMI 25–29.9), and 40% had severe comorbidity (CCI ≥ 5). Active treatment was initiated for 65% of tumours, ranging from 44% to 85% between hospitals (*p* < 0.001). The types of treatment modalities used differed significantly between hospitals. This variation persisted after stratifying for comorbidity and tumour complexity. Independent factors associated with active treatment were Charlson comorbidity index (CCI) (OR = 0.77 95%CI [0.72–0.83], *p* < 0.001) RENAL Nephrometry score (OR = 1.16 95%CI [1.04–1.28], *p* = 0.006), and hospital of diagnosis (*p* < 0.001). After adjustment for case mix, hospital of diagnosis remained a significant predictive factor (*p* < 0.001). Study limitations include potential selection bias and limited generalizability.

**Conclusions:**

Substantial inter‐hospital variation exists in cT1a RCC management, which is not fully explained by patient‐ or tumour characteristics. To reduce unwarranted variation and improve care, transparent care pathways, routine outcome measurement, shared decision‐making and inter‐hospital benchmarking are needed.

**Abbreviations**
AS: active surveillance
BMI: Body Mass Index
CCI: Charlson Comorbidity Index
CWZ: Canisius Wilhelmina Hospital
eGFR: estimated Glomerular Filtration Rate
ICC: intraclass correlation coefficient
IQR: interquartile range
MEC‐U: Medical Research Ethics Committee United
MST: Medisch Spectrum Twente
NCR: Netherlands Cancer Registry (Dutch: Nederlandse Kanker Registratie)
OR: odds ratio
PN: partial nephrectomy
RCC: renal cell carcinoma
RN: radical nephrectomy
RT: radiotherapy
SD: standard deviation
SDM: shared decision‐making
SRM: small renal mass
TA: thermal ablation
VBHC: value‐based healthcare
WMO: Medical Research Involving Human Subjects Act
WW: watchful waiting

## 1 INTRODUCTION

Renal cell carcinoma (RCC) accounts for 3%–5% of all new cancer cases in Europe each year and is therefore the sixth most frequently diagnosed cancer.^1^ The number of new RCC cases in the Netherlands is expected to increase by 31% in the next decade, as risk factors such as hypertension and obesity are expected to rise further.^2^ In addition, the rising incidence is attributable to the more frequent incidental detection of small renal masses (SRMs) during imaging performed for unrelated reasons.^3^ In the Netherlands, more than half of the RCC diagnoses are cT1 tumours (located only in the kidney, <7 cm), of which 36% are cT1a tumours (≤4 cm).^4^

Treatment options for RCC depend on disease stage, tumour location, histopathology, patient factors (e.g., comorbidity and obesity) and patient preferences for treatment. The standard of care for the treatment of cT1a RCC is partial nephrectomy (PN), but thermal ablation (TA) and active surveillance (AS) are alternatives.^5^ A 12‐year prospective study has recently confirmed the safety and long‐term oncological outcomes of AS.^6^ In addition, radiotherapy (RT) is an emerging therapy for SRMs.^7^, ^8^ For some patients, watchful waiting (WW) or managing symptoms as they occur (best supportive care) are appropriate treatment options. In the Netherlands, the local availability of specific treatment modalities, such as surgery or TA, varies between hospitals. Ideally, the decision on the most appropriate treatment should be based on oncological efficacy, preservation of renal function, treatment‐related complications and patient preferences for appropriate treatment options (e.g. impact on quality of life). In line with the concept of shared decision‐making (SDM), all treatment options should be considered and discussed.^9^, ^10^ Patients should be referred if a particular required treatment modality is not available at the hospital of diagnosis.

A recent study has shown practice variation in the treatment of cT1 RCC in the Netherlands, with treatment strategies influenced by surgical hospital volume.^11^ Variation in treatment is, to some extent, inevitable and even necessary, as each patient has unique characteristics and needs. In some cases, variation may be unjustified.^12^

For example, the range of treatment options available at a given hospital or in a certain region may be a factor. Currently, the treatment options offered to patients may depend on the hospital or region in which they are diagnosed. This could result in patients being over‐ or undertreated, or certain treatment options being withheld. In addition, variation could result in differences in outcomes or care costs between hospitals. Therefore, it is important to identify factors associated with variation in the treatment of cT1a RCC. In this way, unwarranted differences can be identified and reduced. The aim of this study is to analyse treatment variation for cT1a renal cancer in seven large teaching hospitals in the Netherlands. Additionally, we will investigate factors associated with this variation.

## 2 PATIENTS AND METHODS

### 2.1 Study design

This retrospective, multicenter cohort study was performed within a network of seven large nonacademic teaching hospitals in the Netherlands, the Santeon hospital group: St. Antonius Hospital, OLVG, Catharina Hospital, Canisius Wilhelmina Hospital (CWZ), Martini Hospital, Maasstad Hospital and Medisch Spectrum Twente (MST). These hospitals are geographically spread throughout the Netherlands and serve approximately 12% of the Dutch population. These hospitals collaborate to implement value‐based healthcare (VBHC).^13^, ^14^

The Medical Research Ethics Committee United (MEC‐U) in Nieuwegein has confirmed that the Medical Research Involving Human Subjects Act (WMO) did not apply to this study (W24.115). The research protocol for this study was approved by the ‘Santeon Beheercommissie’ (SDB 2024‐012). In addition, each participating hospital approved local feasibility. Pseudonymization was applied to all data for the hospitals involved.

### 2.2 Patient population and data collection

Patients aged ≥18 years diagnosed with cT1a renal cancer from 2019 to 2022 were identified by the Netherlands Cancer Registry (NCR). As the NCR includes both histologically confirmed and clinically diagnosed cases, this study population reflects SRMs that are suspected to be RCC, rather than only confirmed RCC. Patients diagnosed outside the Santeon hospitals were excluded. From the NCR, date of birth, gender, date of diagnosis, hospital of diagnosis, treatment, date of treatment and tumour radius were collected and subsequently checked with the electronic patient record in each participating hospital. Next, complementary patient‐, tumour‐ and treatment characteristics were extracted manually from the electronic patient records. Charlson comorbidity index (CCI) was categorized into three groups: no comorbidity (0), mild/moderate comorbidity (1–4) and severe comorbidity (≥5). RENAL Nephrometry scores were categorized into two groups: low (4–6) and intermediate/high (7–12). Estimated glomerular filtration rate (eGFR) was collected around the date of diagnosis (range 4–8 months prior to treatment). Data were collected and managed using REDCap electronic data capture tools.^15^ In addition, information on the available treatment options in each hospital was obtained from urologists at each hospital. Clinical follow‐up was completed until February 2025.

### 2.3 Outcomes

The primary outcome of this study was the initial treatment received for the cT1a renal cancer. Treatment was categorized into six groups: PN, TA, AS, radical nephrectomy (RN), RT or WW/no treatment. Treatment was further categorized into active treatment, including PN, TA, RN and RT. No active treatment was defined as AS or WW/no treatment.

### 2.4 Statistical analyses

With descriptive analyses, we provided insight into patient‐ and tumour characteristic of the total population and by hospital. Continuous variables were presented as mean ± standard deviation (SD) or median with interquartile range (IQR). Categorical data were presented as frequencies with percentage and compared using Chi‐square tests. Due to the limited sample size, no statistical testing was performed in small groups. Furthermore, treatment distributions across hospitals were visualized. Post hoc analyses were conducted using standardized *z*‐tests to identify significant deviations between observed and expected frequencies for each hospital‐treatment combination.

In addition, we have stratified all analyses by CCI score (mild/moderate vs. severe) and RENAL Nephrometry score (low vs. intermediate/high). Next, analyses were repeated in tumours suitable for PN (RENAL Nephrometry score 4–6, CCI < 5, eGFR ≥ 60). Univariable and multivariable logistic regression was performed to determine which factors were associated with initiating active treatment. Missing values were handled using listwise deletion.

To evaluate how much of the variation in treatment decisions could be attributed to differences between hospitals, we calculated the intraclass correlation coefficient (ICC) using a null multilevel logistic regression model, with hospital included as a random intercept. A multilevel analysis was deemed necessary if the percentage was over 10%.^16^ A *p*‐value <0.05 was reported as a significant difference. Statistical analyses were performed with SPSS version 29.0.

## 3 RESULTS

### 3.1 Tumour characteristics

Overall, 501 patients with 544 cT1a renal cancer tumours were diagnosed at the seven hospitals from 2019 to 2022. A total of 35 patients presented with more than one tumour. Mean age of patients was 66 years, 40% were overweight (body mass index [BMI] 25–29.9), and 40% had severe comorbidity (CCI ≥ 5). Mean eGFR was 69.4 mL/min/1.73 m^2^ and 48% of tumours presented in the left kidney. Mean tumour radius was 2.5 cm and the majority of tumours had a low RENAL Nephrometry score (4–6, *n* = 283, 52%) (Table 1). Patient‐ and tumour characteristics differed minimally between hospitals.

**TABLE 1:** Tumour characteristics in patients with cT1a renal cancer according to hospital site.

	Total (*n* = 544)	Hospital A (*n* = 60)	Hospital B (*n* = 82)	Hospital C (*n* = 45)	Hospital D (*n* = 46)	Hospital E (*n* = 61)	Hospital F (*n* = 92)	Hospital G (*n* = 158)
**Sex (female), no. (%)**	175 (32)	24 (40)	27 (33)	21 (47)	11 (24)	21 (34)	26 (28)	45 (29)
**Mean age at diagnosis, years ± SD**	65.6 ± 11.4	67.8 ± 10.9	67.7 ± 11.2	70.8 ± 9.8	64.7 ± 11.1	63.7 ± 11.0	65.5 ± 12.0	63.4 ± 11.4
**BMI, no. (%)**								
Underweight (<18.5)	4 (1)	1 (2)	‐	‐	1 (2)	1 (2)	1 (1)	1 (1)
Normal (18.5–24.9)	164 (30)	19 (32)	24 (29)	9 (20)	12 (26)	20 (33)	29 (32)	51 (32)
Preobesity (25–29.9)	219 (40)	23 (38)	36 (44)	20 (44)	22 (36)	22 (36)	30 (33)	71 (45)
Obesitas class I (30–34.9)	101 (19)	12 (20)	13 (16)	5 (11)	14 (30)	10 (16)	18 (20)	29 (18)
Obesitas class II (35–39.9)	36 (7)	4 (7)	5 (6)	4 (9)	3 (7)	5 (8)	9 (10)	6 (4)
Obesitas class III (>40)	4 (1)	‐	1 (1)	1 (2)	‐	‐	2 (2)	‐
Missing	16 (3)	1 (2)	3 (4)	6 (13)	3 (5)	3 (5)	3 (3)	‐
**Charlson Comorbidity Index, no. (%)**								
No comorbidity (0)	34 (6)	1 (2)	1 (1)	‐	3 (7)	8 (13)	5 (5)	16 (10)
Mild (1–2)	129 (24)	14 (23)	17 (21)	6 (13)	12 (26)	15 (25)	24 (26)	41 (26)
Moderate (3–4)	162 (30)	18 (30)	28 (34)	9 (20)	20 (44)	17 (28)	21 (23)	49 (31)
Severe (≥5)	219 (40)	27 (45)	36 (44)	30 (67)	11 (24)	21 (34)	42 (46)	52 (33)
**Smoking/history with smoking, no. (%)**								
Yes	313 (58)	30 (50)	50 (61)	21 (47)	19 (41)	36 (59)	60 (65)	97 (61)
No	177 (32)	25 (42)	25 (31)	21 (47)	17 (37)	6 (10)	28 (30)	55 (35)
Missing	54 (10)	5 (8)	7 (9)	3 (7)	10 (22)	19 (31)	4 (4)	6 (4)
**Hypertension, no. (%)**								
Yes	316 (58)	38 (63)	59 (72)	28 (62)	16 (35)	22 (36)	48 (52)	105 (67)
No	185 (34)	20 (33)	19 (23)	16 (36)	24 (52)	13 (21)	44 (48)	49 (31)
Missing	43 (8)	2 (3)	4 (5)	1 (2)	6 (13)	26 (43)	‐	4 (3)
**eGFR, ml/min/1,73m** ^**2**^ **, mean ± SD**	69.35 ± 22.55	63.72 ± 22.5	66.70 ± 20.7	67.56 ± 21.5	77.89 ± 19.6	73.92 ± 22.1	66.43 ± 26.3	70.21 ± 21.5
Missing	*n* = 36	*n* = 13	*n* = 13	*n* = 0	*n* = 1	*n* = 1	*n* = 2	*n* = 6
**Tumour location (left kidney), no. (%)**	261 (48)	29 (48)	35 (43)	24 (53)	25 (54)	26 (43)	37 (40)	85 (54)
**Tumour radius (mm), mean ± SD**	24.8 ± 8.6	26.0 ± 8.3	23.7 ± 8.1	23.4 ± 9.2	27.3 ± 7.6	24.0 ± 8.2	26.3 ± 8.5	24.0 ± 9.2
**RENAL Nephrometry score, no. (%)**								
Low (4–6)	283 (52)	41 (68)	42 (51)	21 (47)	20 (44)	33 (54)	45 (49)	81 (51)
Intermediate (7–9)	242 (45)	18 (30)	38 (46)	20 (44)	26 (57)	23 (38)	43 (47)	74 (47)
High (10–12)	19 (4)	1 (2)	2 (2)	4 (9)	‐	5 (8)	4 (4)	3 (2)

Abbreviations: BMI, body mass index; eGFR, estimated glomerular filtration rate; SD, standard deviation.

Table S1 provides an overview of the treatment modalities available at each hospital (Supporting Information: Appendix I). There were few differences in the treatment options offered by the hospitals. None of the hospitals offered RT, and Hospital C did not offer surgery in‐house.

### 3.2 Active versus no active treatment

**FIGURE 1:**
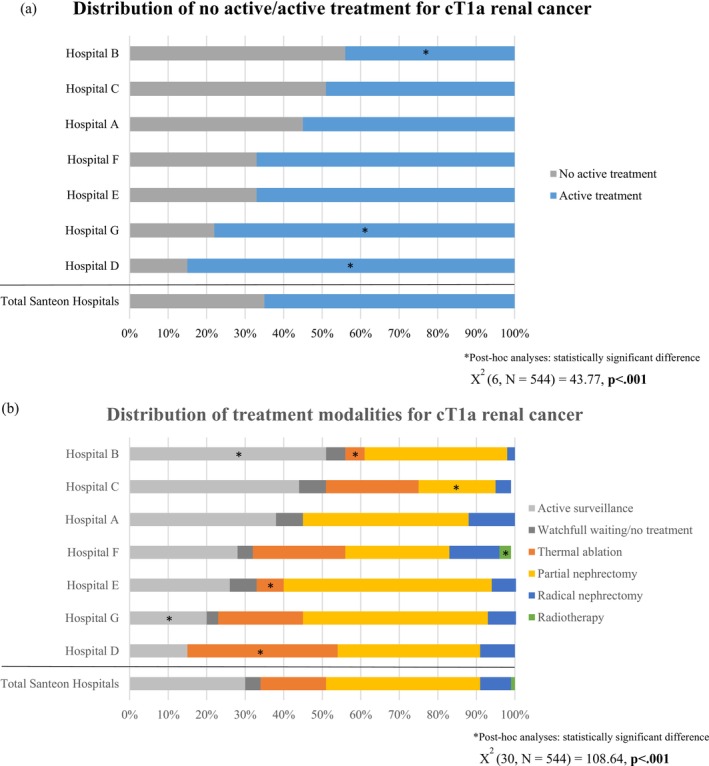
(A,B) Distribution of treatment modalities for cT1a renal cancer across hospitals, categorized by active/no active treatment (A) and specific treatment modalities (B).

Figure 1 displays the distribution of active versus nonactive treatment across hospitals. Overall, 65% of tumours were actively treated and this percentage varied largely across the seven hospitals (44%–85%) (Figure 1A). Post hoc analysis revealed that Hospital B performed significantly *fewer* active treatments than expected (*z* = −2.4). Hospitals D and G had significantly *more* patients receiving active treatment than expected (*z* = −2.2 and *z* = −2.7, respectively) (*p* < 0.05).

### 3.3 Specific treatment modalities

Significant differences were also observed between hospitals when specific treatment modalities were considered (Figure 1B). PN and TA were found to vary between hospitals from 20% to 54% and 0% to 39%, respectively. AS was chosen in 15%–51% of tumours and RN was performed in 2%–13% of tumours. Initial treatment rates for specific treatment modalities are presented in Table S2 (Appendix II). Post hoc analyses showed that tumours in patients diagnosed in Hospitals A or E were *less often* managed with TA than expected (*z* = −3.2 and *z* = −2.0, respectively). Tumours diagnosed in Hospital B were managed significantly *more* with AS (*z* = 3.4) and significantly less with TA (*z* = −2.7). Patients with tumours diagnosed in Hospital C were *less often* managed with PN than expected (*z* = −2.1), while tumours in patients diagnosed in Hospital D were managed *more often* with TA (*z* = 3.6). Tumours in patients diagnosed in Hospital F were significantly *more likely* to be treated with radiotherapy, as these patients were referred to a regional hospital for this treatment (*z* = 3.5). In Hospital G, tumours were *less often* managed with AS than expected (*z* = −2.4) (*p* < 0.05).

After stratification by comorbidity and tumour complexity, significant variation in treatment across hospitals remained, as shown in Figure S1 (Appendix III) (*p* < 0.001). In tumours from patients eligible for PN, the gold standard of therapy, 61% received PN, with hospital rates ranging from 43 to 71% (Figure S2, Appendix IV).

### 3.4 Factors effecting active treatment

Univariable logistic regression analysis revealed that CCI and RENAL Nephrometry score were independent significant predictive factors for receiving active treatment (Odds ratio (OR) = 0.77 95% CI [0.72–0.83], *p* < 0.001 and OR = 1.16 95% CI [1.04–1.28], *p* = 0.006, respectively). This indicates that tumours in patients with fewer comorbidities and more anatomically complex tumours were more likely to undergo active treatment. In addition, hospital of diagnosis was significantly associated with whether or not tumours were treated with active treatment (*p* < 0.001), indicating that treatment approach varied by hospital of diagnosis. Lastly, eGFR prior to treatment was a significant predictor of receiving active treatment (OR = 1.02 95% CI [1.00–1.02], *p* < 0.001).

Multivariable logistic regression analysis showed that the hospital of diagnosis remained significantly associated with active or no active treatment, even after correction for CCI, RENAL Nephrometry score, and eGFR (*p* < 0.001).

With an ICC of 0.081, it was found that around 8% of the total variation in treatment decisions could be attributed to the hospital of diagnosis. As this was below the predefined threshold of 10%, no further multilevel analysis was conducted.

## 4 DISCUSSION

This study evaluated variation in the management of cT1a renal cancer in seven Dutch teaching hospitals. Significant differences in treatment distributions were found across hospitals, and differences persisted after stratifying for comorbidity and tumour complexity. Multivariable analyses showed that after case mix adjustment, hospital of diagnosis remained a significant factor associated with initiating active versus no active treatment.

First, although this study shows a treatment distribution comparable to previous studies on cT1 RCC,^11^, ^17^ we observed substantial differences in treatment distributions across hospitals, even after adjustment for patient and tumour factors. Instead, our findings suggest the presence of unwarranted practice variation, whereby patients might not always receive care that aligns with their personal values and preferences.^12^ This is of particular concern in the context of cT1a RCC, where several treatment options, including AS, TA and PN, are considered appropriate. In the absence of well‐defined, evidence‐based pathways, treatment decision‐making may differ across hospitals. Variation in practice is not a new phenomenon within healthcare and has been observed in many different contexts.^12^ Nevertheless, it is not necessarily considered problematic. However, the differences observed in our study are undesirable, as they suggest that treatment decisions depend on institutional factors, such as the availability of specific modalities, expertise or clinician preferences. Our findings align with recent UK research, showing that hospitals with greater experience and higher surgical volumes are more likely to perform PN, regardless of tumour complexity.^18^ Similarly, a recent study showed that the primary urologist was the key factor in determining cT1a RCC management decisions.^19^ Our findings suggests that institutional factors may heavily influence treatment decisions, potentially resulting in under‐ or overtreatment and affecting patients' quality of life and clinical outcomes. A key objective of accessible healthcare is to ensure that patients receive the same standard of care, regardless of where they are diagnosed.^20^ Therefore, it is essential to understand the causes of this variation in order to promote consistency in care.

Second, this analysis was conducted as part of the wider VBHC implementation in the Santeon network, aiming to improve RCC care.^21^ To translate our findings into sustainable improvements, the next step in VBHC implementation is required: transitioning from individual, hospital‐level initiatives to organized, collective learning. While individual participating hospitals have implemented VBHC principles, a structured framework for shared learning has been lacking. However, this structured framework has been successfully established for 16 medical conditions included in the Santeon ‘Better Together’ programme, with the number of included conditions continuing to grow. This programme enables VBHC implementation by providing data insights, supporting multidisciplinary improvement teams and facilitating collaboration.^13^, ^14^ We therefore recommend incorporating RCC into this programme to support standardized care pathways, inter‐hospital benchmarking and the systematic implementation of improvements in line with the Santeon approach to standardizing care. Moreover, similar efforts have been done for prostate cancer, where similar patterns have been observed in prostate cancer management across Santeon hospitals, with variations in treatment, particularly among patients with localized prostate cancer.^22^ Because of these findings, the Santeon hospitals have developed a uniform care pathway based on standardized diagnostics, aiming for accurate diagnoses and appropriate treatment.

This study has some limitations. First, the study population was derived from seven Dutch teaching hospitals and may not be representative of national practice. Nevertheless, their regional distribution is likely to improve the external validity of our findings. In addition, hospitals differed in treatment availability. Most had made regional agreements with other hospitals to streamline RCC care. For instance, at Hospital C, patients requiring active treatment were often referred to a regional hospital specialized in RCC care not included in this study. This reflects real‐world organizational variation, which should be considered when interpreting the findings. Consequently, cases involving active treatment may have been underrepresented in that hospital's data. Secondly, while the NCR includes both histologically and clinically diagnosed RCC cases, tumours without histological confirmation that are not actively treated, and with no clear clinical diagnosis of malignancy (e.g. those under AS/WW without diagnostic certainty), may be underrepresented. Treatment patterns for this subgroup could not be fully evaluated. Furthermore, a lack of data on patient outcomes and preferences limited insight into treatment rationale. Additionally, lacking data on clinician‐related factors (e.g. experience) may influence treatment decision‐making and contribute to variation. Finally, the retrospective design resulted in missing data, leading to exclusions in the multivariable analysis and potentially affecting the robustness of the findings. Nevertheless, observed hospital differences remain relevant, reflecting variations not fully explained by case mix. To address these limitations, a nationwide RCC registry has recently been initiated to enable more comprehensive analyses of practice variation and patient‐centred outcomes.^23^ Despite limitations, this study offers insights from a large multicenter cohort, highlighting substantial inter‐hospital variation in cT1a renal cancer management and the need for standardized care pathways. Future research should explore how institutional factors influence treatment decisions and outcomes, to help reduce unwarranted variation and improve collaborative learning in optimizing RCC management.

## 5 CONCLUSIONS

This study demonstrates substantial variation between hospitals in cT1a renal cancer management, which cannot be fully explained by differences in patient comorbidity or tumour complexity. Treatment decisions were significantly associated with the hospital of diagnosis, suggesting that institutional factors play a key role. In order to reduce unwarranted variation and improve the consistency and quality of care, transparent care pathways, standardized outcome measurement, shared decision‐making and inter‐hospital benchmarking are essential.

## AUTHOR CONTRIBUTIONS

Study concept and design: Cato Caroline Bresser, Paul Bastiaan van der Nat, Hilin Yilidrim, Lea Magdalena Dijksman, Pepijn Dione Polm, Mirjam Marjolein Garvelink and Harm Hubertus Edmundus van Melick. Acquisition of data: Cato Caroline Bresser, Hilin Yilidrim, Bart Jean Pieter Kersten, Hendrik Johannes Josephus Leenarts, Katja Karen and Helmi Aben. Analysis andinterpretation of data: Cato Caroline Bresser, Paul Bastiaan van der Nat, Hilin Yilidrim, Katja Karen Helmi Aben, Lea Magdalena Dijksman, Pepijn Dione Polm, Mirjam Marjolein Garvelink and Harm Hubertus Edmundus van Melick. Drafting of the manuscript: Cato Caroline Bresser. Critical revision of the manuscript for important intellectual content: Paul Bastiaan van der Nat, Hilin Yilidrim, Bart Jean Pieter Kersten, Hendrik Johannes Josephus Leenarts, Katja Karen Helmi Aben, Patricia Jeannelle Zondervan, Lea Magdalena Dijksman, Pepijn Dione Polm, Mirjam Marjolein Garvelink, Harm Hubertus Edmundus van Melick, Wouterus Antonius Scheepens, Sybren Dijkstra, Marit Josephine Yska, Daphne Luijendijk, Marino Asselman and Annebeth Evelien Cathelijn Ruiter. Statistical analysis: Cato Caroline Bresser. Obtaining funding: Cato Caroline Bresser, Paul Bastiaan van der Nat, Lea Magdalena Dijksman, Mirjam Marjolein Garvelink, Harm Hubertus Edmundus van Melick. Administrative, technical or material support: None. Supervision: Paul Bastiaan van der Nat, Mirjam Marjolein Garvelink and Harm Hubertus Edmundus van Melick. Other: None.

## ACKNOWLEDEGMENTS

The authors thank the registration team of the Netherlands Comprehensive Cancer Organisation (IKNL) for the collection of data for the Netherlands Cancer Registry as well as IKNL staff for scientific advice. This research has been made possible in part by the support of the St. Antonius Onderzoeksfonds.

## CONFLICT OF INTEREST STATEMENT

The authors declare that they have no known competing financial interests or personal relationships that could have appeared to influence the work reported in this paper.

## Supporting information


**Table S1.** Available treatment modalities performed at each hospital site (2019–2022).
**Table S2.** Initial treatment ranges in % of tumours for cT1a renal cancer according to hospital site for all tumours and stratified by comorbidity, tumour complexity, and age.
**Figure S1. a, b, c, d.** Distribution of treatment modalities for cT1a renal cancer across hospitals, stratified by mild/moderate comorbidity (CCI < 5) (a) and severe comorbidity (CCI ≥ 5) (b), and by low (4–6) (c) and intermediate/high (7–12) RENAL Nephrometry score (d).
**Figure S2.** Distribution of treatment modalities for cT1a renal cancer across hospitals, only including tumours in patients eligible for partial nephrectomy.

## Data Availability

CB had full access to all the data in the study and takes responsibility for the integrity of the data and the accuracy of the data analysis.
