# Supplementary material for: Hospital variation in the treatment of cT1a renal cancer
Source: BJUI Compass. 2026 Apr 9;7(4):e70130. doi: 10.1002/bco2.70130 (PMC13063115; doi:10.1002/bco2.70130)
Supplement: Supplementary file 1 — Table S1. Available treatment modalities performed at each hospital site (2019–2022). Table S2. Initial treatment ranges in % of tumours for cT1a renal cancer according to hospital site for all tumours and stratified by comorbidity, tumour complexity, and age. Figure S1. a, b, c, d. Distribution of treatment modalities for cT1a renal cancer across hospitals, stratified by mild/moderate comorbidity (CCI < 5) (a) and severe comorbidity (CCI ≥ 5) (b), and by low (4–6) (c) and intermediate/high (7–12) RENAL Nephrometry score (d). Figure S2. Distribution of treatment modalities for cT1a renal cancer across hospitals, only including tumours in patients eligible for partial nephrectomy. [file BCO2-7-e70130-s001.docx]

**Appendix I: Supplementary Table S1**

**Supplementary Table S1.** Available treatment modalities performed at each hospital site (2019-2022)

| **Hospital** | **Partial nephrectomy** | **Radical nephrectomy** | **Thermal ablation** | **Radiotherapy** | **Active surveillance** | **Watchfull waiting/no treatment** |
| --- | --- | --- | --- | --- | --- | --- |
| **A** | **✓** | **✓** | **✓**^1^ | ✘ | **✓** | **✓** |
| **B** | **✓** | **✓** | **✓** | ✘ | **✓** | **✓** |
| **C** | ✘^2^ | ✘^2^ | **✓** | ✘ | **✓** | **✓** |
| **D** | **✓** | **✓** | **✓** | ✘ | **✓** | **✓** |
| **E** | **✓^3^** | **✓** | **✓** | ✘ | **✓** | **✓** |
| **F** | **✓** | **✓** | **✓** | ✘ | **✓** | **✓** |
| **G** | **✓** | **✓** | **✓** | ✘ | **✓** | **✓** |

^1^TA performed since June 2022

^2^Did not offer surgery in-house, but as part of a partnership in the region

^3^Robot-assisted partial nephrectomy offered since 2021; prior to that, only open partial nephrectomy was performed in-house.

**Appendix II: Supplementary Table S2**

**Supplementary Table S2.** Initial treatment ranges in % of tumors for cT1a renal cancer according to hospital site for all tumors and stratified by comorbidity, tumor complexity, and age.

|  | **Total**  n=544 | **CCI<5**  n=325 | **CCI≥5**  n=219 | **RENAL 4-6**  n=283 | **RENAL 7-12**  n=261 |
| --- | --- | --- | --- | --- | --- |
| **Active surveillance** | 15-51 | 14-41 | 18-64 | 10-52 | 17-55 |
| **Partial nephrectomy** | 20-54 | 33-68 | 12-29 | 24-58 | 17-53 |
| **Radical nephrectomy** | 2-13 | 4-11 | 0-19 | 0-11 | 5-21 |
| **Thermal ablation** | 0-39 | 0-34 | 0-55 | 0-40 | 0-39 |
| **Radiotherapy** | 0-3 | 0-2 | 0-5 | 0-2 | 0-4 |
| **Watchfull waiting/no treatment** | 3-7 | 0-4 | 0-14 | 0-10 | 0-7 |

*CCI* Charlson Comorbidity Index

**Appendix III: Supplementary Figure S1**

**
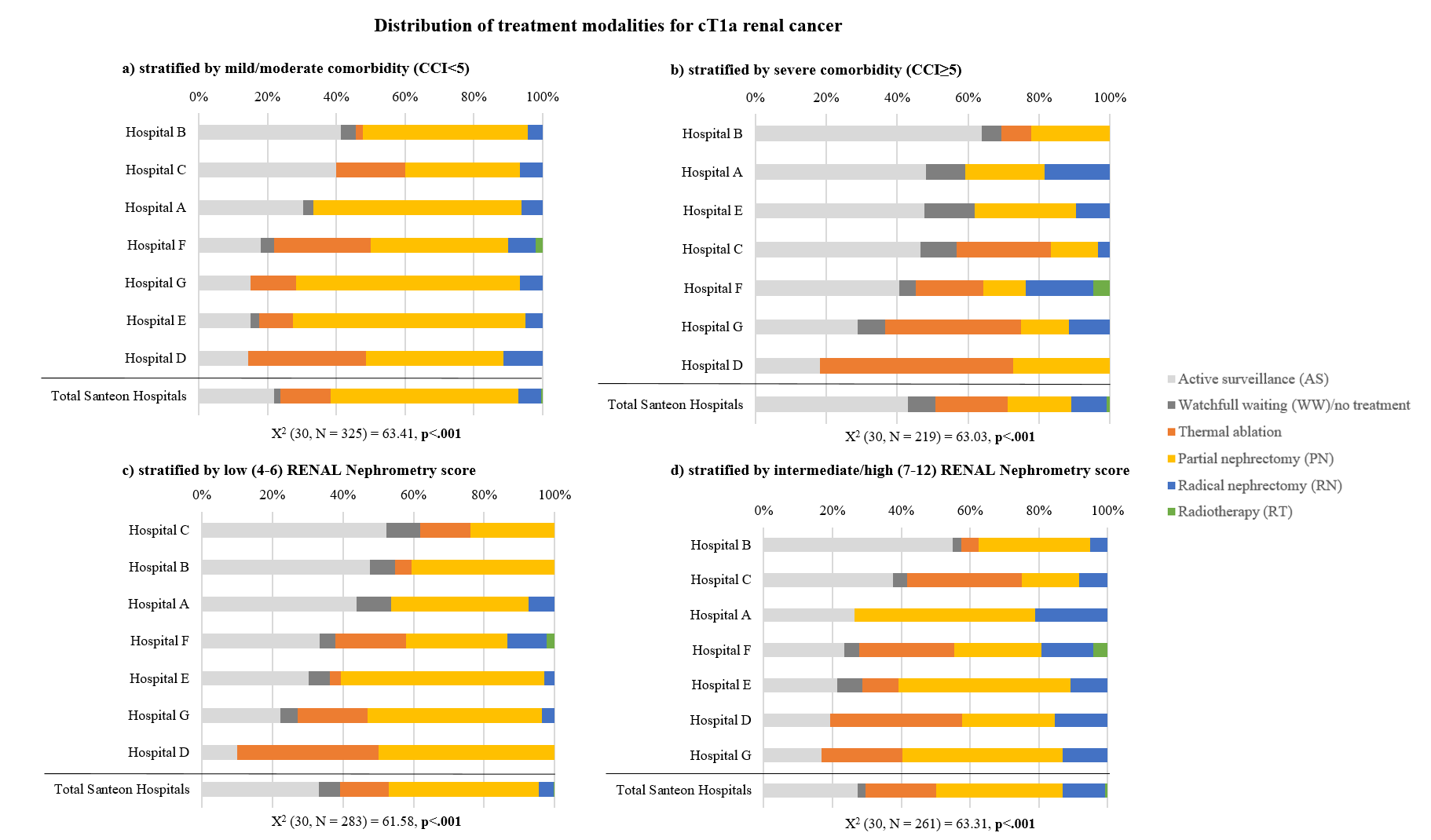
**

**Supplementary Figure S1. a, b, c, d.** Distribution of treatment modalities for cT1a renal cancer across hospitals, stratified by mild/moderate comorbidity (CCI<5) (a) and severe comorbidity (CCI≥5) (b), and by low (4-6) (c) and intermediate/high (7-12) RENAL Nephrometry score (d).

**Appendix IV: Supplementary Figure S2**

**Supplementary Figure S2.** Distribution of treatment modalities for cT1a renal cancer across hospitals, only including tumors in patients eligible for partial nephrectomy.
